# Compatible Solid Polymer Electrolyte Based on Methyl Cellulose for Energy Storage Application: Structural, Electrical, and Electrochemical Properties

**DOI:** 10.3390/polym12102257

**Published:** 2020-10-01

**Authors:** Shujahadeen B. Aziz, Iver Brevik, Muhamad H. Hamsan, M. A. Brza, Muaffaq M. Nofal, Aziz M. Abdullah, Sarkawt Rostam, Shakhawan Al-Zangana, Saiful K. Muzakir, Mohd F. Z. Kadir

**Affiliations:** 1 Hameed Majid Advanced Polymeric Materials Research Lab., Physics, College of Science, University of Sulaimani, Qlyasan Street, Sulaimani 46001, Iraq; 2 Department of Civil Engineering, College of Engineering, Komar University of Science and Technology, Sulaimani 46001, Iraq; 3 Department of Energy and Process Engineering, Norwegian University of Science and Technology, N-7491 Trondheim, Norway; 4 Institute for Advanced Studies, University of Malaya, Kuala Lumpur 50603, Malaysia; hafizhamsan93@gmail.com; 5 Manufacturing and Materials Engineering Department, Faculty of Engineering, International Islamic University of Malaysia, Kuala Lumpur 50603, Malaysia; mohamad.brza@gmail.com; 6 Department of Mathematics and General Sciences, Prince Sultan University, P. O. Box 66833, Riyadh 11586, Saudi Arabia; muaffaqnofal@gmail.com; 7 Department of Physics, College of Education, Charmo University, Peshawa Street, Chamchamal, Sulaimani 46001, Iraq; aziz.abdullah@charmouniversity.org; 8 Department of Mechanical Engineering/Production, College of Engineering, Sulaimani Polytechnic University, Sulaimani 46001, Iraq; sarkawt.rostam@spu.edu.iq; 9 Department of Physics, College of Education, University of Garmian, Kalar 46021, Iraq; shakhawan.al-zangana@garmian.edu.krd; 10 Material Technology Program, Faculty of Industrial Sciences & Technology, Universiti Malaysia Pahang, Lebuhraya Tun Razak, Gambang, Kuantan 43600, Malaysia; saifful@ump.edu.my; 11 Centre for Foundation Studies in Science, University of Malaya, Kuala Lumpur 50603, Malaysia; mfzkadir@um.edu.my

**Keywords:** green polymer electrolytes, sodium salt, XRD study, impedance analysis, energy storage device

## Abstract

Compatible green polymer electrolytes based on methyl cellulose (MC) were prepared for energy storage electrochemical double-layer capacitor (EDLC) application. X-ray diffraction (XRD) was conducted for structural investigation. The reduction in the intensity of crystalline peaks of MC upon the addition of sodium iodide (NaI) salt discloses the growth of the amorphous area in solid polymer electrolytes (SPEs). Impedance plots show that the uppermost conducting electrolyte had a smaller bulk resistance. The highest attained direct current DC conductivity was 3.01 × 10^−3^ S/cm for the sample integrated with 50 wt.% of NaI. The dielectric analysis suggests that samples in this study showed non-Debye behavior. The electron transference number was found to be lower than the ion transference number, thus it can be concluded that ions are the primary charge carriers in the MC–NaI system. The addition of a relatively high concentration of salt into the MC matrix changed the ion transfer number from 0.75 to 0.93. From linear sweep voltammetry (LSV), the green polymer electrolyte in this work was actually stable up to 1.7 V. The consequence of the cyclic voltammetry (CV) plot suggests that the nature of charge storage at the electrode–electrolyte interfaces is a non-Faradaic process and specific capacitance is subjective by scan rates. The relatively high capacitance of 94.7 F/g at a sweep rate of 10 mV/s was achieved for EDLC assembly containing a MC–NaI system.

## 1. Introduction

Green biopolymers degrade naturally, whereas synthetic or human-made polymers are relatively non-biodegradable [1]. The most abundant green polymer in nature is cellulose; however, pure cellulose cannot be dissolved in water [2], thus, modification is needed. For example, inclusion of methyl chloride to the cellulose (methylation) will produce methyl cellulose (MC). MC is known to have a reasonable price and is eco-friendly as well as having acceptable film forming properties such as transparency and superior mechanical and electrical properties [3]. Cations can form interactions with O_2_ atoms of MC over dative bonds. MC contains functional groups, for instance, C–O–C, O–H, and O–CH_3_ groups have lone pair electrons that are in charge for the ionic conduction [4]. Additionally, it is an amorphous polymer that has a relatively high glass transition temperature (T_g_), ranging from 184 to 200 °C [5].

The present studies have been conquered by lithium-ion based systems for their potential use in solid-state supercapacitors, with one scarce study that used polymer electrolytes based on sodium ion complexed films [6,7]. The primary advantage of sodium salt employment is its low atomic mass and accessibility in abundance at an inexpensive cost compared to lithium salt. The NaI salt selection is mostly due to its ready availability, lower cost, and lower toxicity [8,9,10]. Other than that, a relative study on the effect of lithium and sodium triflate in a polyacrylonitrile (PAN) host conducted by Osman et al. [11] recognized that the sodium-based electrolyte system accomplished upper ionic conductivity at room temperature.

Supercapacitors are energy storage devices characterized by long life-cycle and high power density. They are used for stationary and mobile applications, for example, power stabilization in electrical grids or regenerative braking in hybrid electric vehicles [12,13,14]. There are three kinds of supercapacitors (SCs): pseudocapacitors (PCs), electric double-layer capacitors (EDLCs), and hybrid capacitors. For EDLCs, the charges are stored and released at the electrolyte/electrode interfaces. For PCs, the charges are stored mostly at the expense of the reversible Faradaic redox reaction of active material in the electrodes [15]. Nowadays, EDLCs have been studied and fabricated using various electrodes (e.g., carbon electrodes including activated carbon (AC), graphite, carbon nanogel, and carbon nanotubes (CNTs), etc.). EDLC can be considered as a good replacement for conventional batteries, especially in low energy density applications [16,17]. AC is the most widely used activation material in the application of EDLC due to its easy preparation, large surface area, and low-cost. The surface area of AC is around 2500 m^2^/g. As the energy storage process of EDLC is through the adsorption/desorption or non-Faradaic process, a big surface area electrode is essential [18]. The routine of EDLC can be superior either through the modification of the electrolytes or electrodes. Current studies have shown that polymer electrolytes (PEs) are suitable for electrochemical devices due to their features including solvent free, leakage free, light weight, easily forms thin films, easy handling, and widespread electrochemical windows compared to commercial liquid electrolyte counterparts [19,20]. Broad studies have been conducted on ammonium salts interacted with MC for fabrication of PEs [21,22,23], however, little effort has been made toward PEs including sodium salts. Sodium is available in richness at a cheaper cost than lithium. In addition, the softness of the materials makes it easier to attain and maintain contact with other components in batteries [24]. In this work, MC was chosen as the host polymer while ions were provided by the addition of NaI at various concentrations. The best conducting sample will be used in the fabrication of an AC-based EDLC.

## 2. Experimental Details

### 2.1. Materials and Sample Preparation

**Figure 1:**
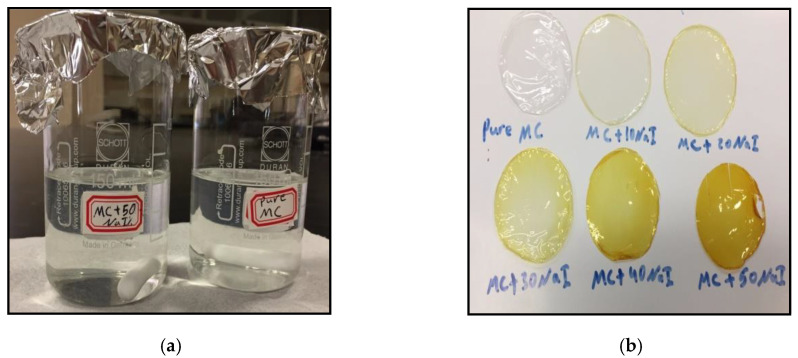
(**a**) Pure MC and doped MC aqueous solution and (**b**) pure MC and doped MC SPE films.

In the preparation of methyl cellulose (MC)-based solid polymer electrolytes (SPEs) incorporated with various quantities of sodium iodide (NaI), the solution cast methodology was used. Both MC and NaI as raw materials were purchased from Sigma-Aldrich (Kuala Lumpur, Malaysia). The first series of solutions were prepared by dissolving 1 g of MC in 80 mL distilled water with stirring using a magnetic stirrer for several hours until an identical aqueous solution was obtained at room temperature, as shown in Figure 1a. A second series of solutions consisting of 10 to 50 wt.% of NaI salt was prepared and then added into the first series of solution followed by continuous stirring until clear real solutions were obtained (Figure 1a). The MC doped with NaI samples were coded as MCSPE1, MCPE2, MCSPE3, MCSPE4, and MCSPE5, which corresponded to10, 20, 30, 40, and 50 wt.% of NaI, respectively. These series of solutions were cast into a number of Petri dishes and then left to make a dry film that was free of solvent at room temperature, as shown in Figure 1b. Afterwards, for further drying, the SPE films (Figure 1b) were put into a desiccator having silica gel. The components of the SPE-based on MC and their corresponding concentrations are presented in Table 1.

### 2.2. X-Ray Diffraction

For XRD acquisitions, a Siemens D5000 x-ray diffractometer (Panalytical Ltd., Malvern, UK) under certain electric operating conditions (40 mA and 40 kV) was applied. A beam of monochromatic CuKα x-radiation of wavelength (λ = 1.5406 Å) and at the sideways angles in the range of 10° ≤ 2θ ≤ 80° with a step size of 0.1° was scanned over each sample.

### 2.3. Electrical Impedance Spectroscopy (EIS)

To study the electrochemical properties of materials that have been utilized in electrochemical devices, complex impedance spectroscopy (CIS) is an appropriate technique [25]. It provides invaluable information about the electrical properties at the interfacial area between the electronically conducting electrode and electrolyte that are in contact with each other. Prior to impedance measurements, the SPE films were cut into small discs (2 cm in diameter) and then inserted between two stainless steel electrodes using spring pressure. The impedance measurements were carried out using a hyphenated HIOKI 3531 Z Hi-tester (Hioki, Nagano, Japan) with a computer in the frequency range of 50 Hz to 5000 kHz at ambient temperature. Both real (*Z′*) and imaginary (*Z″*) parts of the impedance spectra of the Nyquist plot were controlled by HIOKI LCR software (Hioki, Nagano, Japan). From the intercept of the plot with the real impedance axis, the bulk resistance (*R_b_*) can be obtained. From the *R_b_*, one can calculate the conductivity using the following relationship [25]:

(1) $$\sigma_{dc}=\left(\frac{1}{R_{b}}\right)\times \left(\frac{t}{A}\right)$$

where *t* and *A* are the film thickness and surface area, respectively.

## 3. Electrochemical Characterization

### 3.1. Transference Number Measurement (TNM) Analysis

Both ion and electron transference number (TNM) were measured using V&A Instrument DP3003 (V&A Instrument, Shanghai, China) digital DC power supply. The measurements were started by placing the relatively high conducting electrolyte in a Teflon holder containing stainless steel electrodes. The cell perturbation was performed by applying 0.2 V where both ions and electrons start moving. All these measurements were carried out at room temperature and a relative humidity of ~50%.

### 3.2. Linear Sweep Voltammetry (LSV) Analysis

To find the maximum potential that the electrolytes in this study started to decompose, linear sweep voltammetry (LSV) was conducted using a Digi-IVY DY2300 potentiostat (V&A Instrument, Shanghai, China). The electrolyte was inserted between two stainless steel electrodes of a Teflon holder with applied scan rate of 10 mV/s. The temperature for LSV analysis was kept at 25 °C while the relative humidity of the room was maintained at ~50%.

### 3.3. Fabrication and Characterization of EDLC

A total of 3.25 g of activated carbon (AC) was dried and then mixed with 0.25 g of carbon black (CB). The ball miller (Changsha Yonglekang Equipment Co., Ltd., Changsha, Hunan, China) speed was kept constant at 500 revolution/min for 20 min. To a previous solution, a solution of 0.5 g of polyvinylidene fluoride (PVdF) (Sigma-Aldrich, Kuala Lumpur, Malaysia) and 15 mL of N–methyl pyrrolidone (NMP) (Sigma-Aldrich, Kuala Lumpur, Malaysia) was added, producing a completely dissolved AC–CB black thick solution. This black thick solution was coated on cleaned aluminum foil with acetone using a scalpel blade.

The coated aluminum foils were dried in an oven at 60–70 °C for 1 h. To ensure dryness, the coated aluminum foils were placed into a desiccator. The electrodes were obtained from the films by cutting a geometric area of 2.01 cm^2^. The relatively high electrolyte as a separator was sandwiched between two AC electrodes and packed in a CR2032 coil cell. The EDLC assembly was tested using cyclic voltammetry (CV) at different scan rates. The area of the capacitive loop of the CV was obtained from the origin 9 software (OriginLab Corporation 8.5) using an integration function and the specific capacitance of the EDLC was calculated from the following relationship:

(2) $$C=\int_{V_{l}}^{V_{u}}\frac{I\left(V\right)dV}{2mS_{r}\left(V_{2}-V_{1}\right)}$$

where *m* is the mass of active material (activated carbon) where ions form a charge-double layer and *S_r_* is the scan rate.

## 4. Results and Discussion

### 4.1. X-Ray Diffraction (XRD) Study

**Figure 2:**
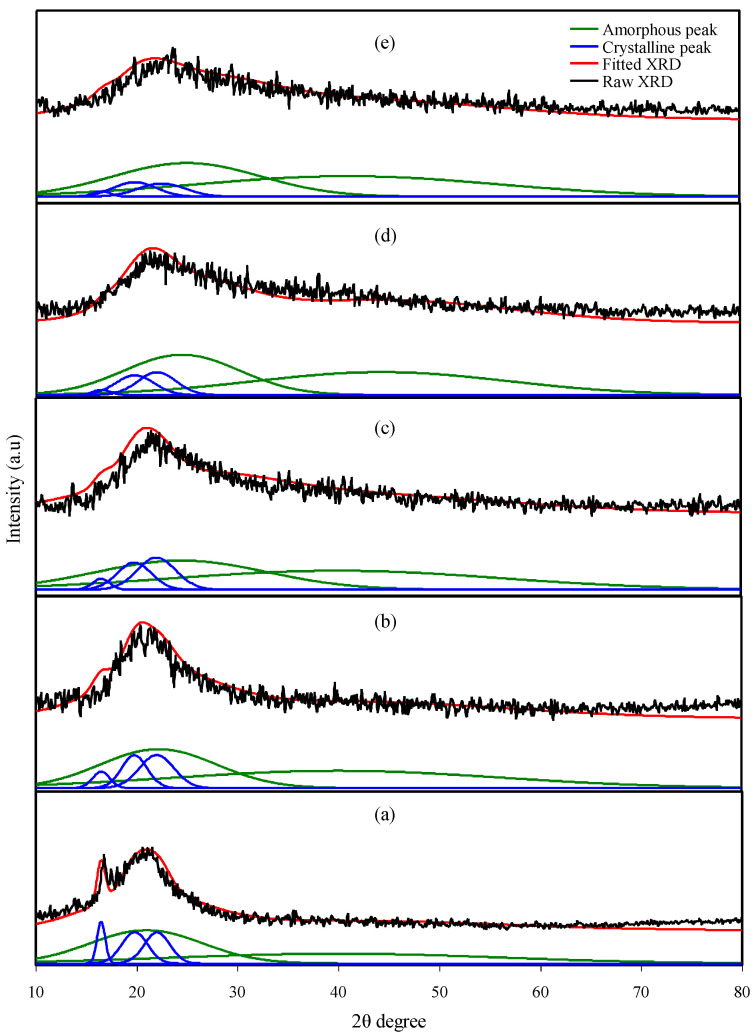
X-ray diffraction (XRD) spectra for (**a**) MCSPE1, (**b**) MCSPE2, (**c**) MCSPE3, (**d**) MCSPE4, and (**e**) MCSPE5 electrolyte films.

**Figure 3:**
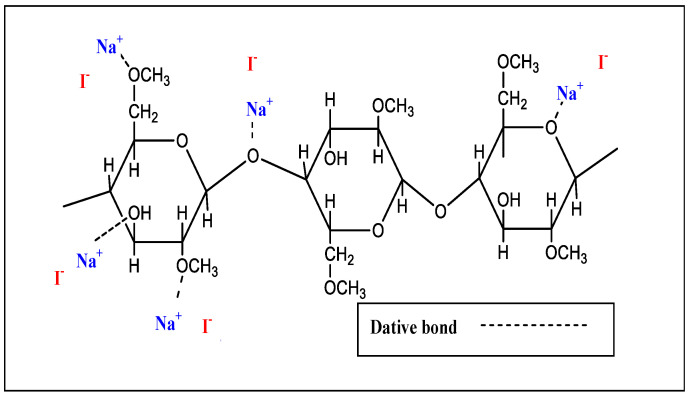
Possible interactions between the NaI and MC matrix through thee dative bond.

All XRD patterns of the MC electrolyte samples are shown in Figure 2. It is well-known that a broad crystalline hump centered at 2θ = 19~21° resulted from strong intermolecular hydrogen bonding along a short distance order of the chains in the MC polymer [26,27]. Earlier work has shown that there is not only a broad hump at 2θ = 19~21°, but also several weak peaks centered at 2θ equal to 8° and 21° are features of pure MC [28,29]. In fact, hydrogen bonding is made from the interaction between electron-deficient hydrogen and high electron density atoms (high electronegative atoms), such as –O and –N. The intermolecular hydrogen bonding can be stated as X–H….Y that X and Y are high electronegative atoms containing electron pairs [30]. MC has functional groups of C–O–C, O–H, and O–CH_3_. Cations create interactions with the oxygen atoms of MC through dative bonds [4]. Figure 3 shows the chemical structure and possible interactions between the NaI and MC matrix through dative bonds.

To determine the degree of crystallinity (*X_c_*), it is essential to deconvolute the XRD spectra of the samples to find the area of the amorphous and crystalline peaks [31]. *X_c_* was calculated using Equation (3) [32],

(3) $$Xc=\frac{A_{C}}{A_{T}}\times 100\%$$

where *A_C_* and *A_T_* are the area of crystalline peaks and total area of amorphous and crystalline peaks, individually. The deconvoluted XRD spectra of MC:NaI electrolytes are indicated in Figure 2a–e. It is important to see that the *X_c_* is decreased upon the inclusion of further NaI salt (see Table 2). It is vital to see that the intensity of the XRD peaks is noticeably decreased. On the basis of earlier study, salt can destroy the hydrogen bonding among the polymer chains due to electrostatic interaction formed between the cations of the salt and the functional groups of the polymer [33]. The increment in amorphous structure in the polymer could be related to the disruption of the crystalline phase [34]. The *Xc* of the MCSPE1 system was 22.38. The lowest *Xc*, which was 10.66, was obtained for MCSPE5. This indicates that MCSPE5 was the most amorphous system. The amorphous phase dominance increased polymer backbone segmental motion, which raised the conductivity and transportation of ions [18]. Compared to MCSPE1, the *Xc* in the electrolyte systems was considerably decreased (see Table 2). Hamsan et al. [21] used the deconvolution method for XRD spectra to determine the *Xc* for the system of MC–PS–NH_4_NO_3_–glycerol-based electrolytes. The *Xc* of their system was 10.39, which is quite close to the present work for the highest salt concentration.

### 4.2. Impedance Study

**Figure 4:**
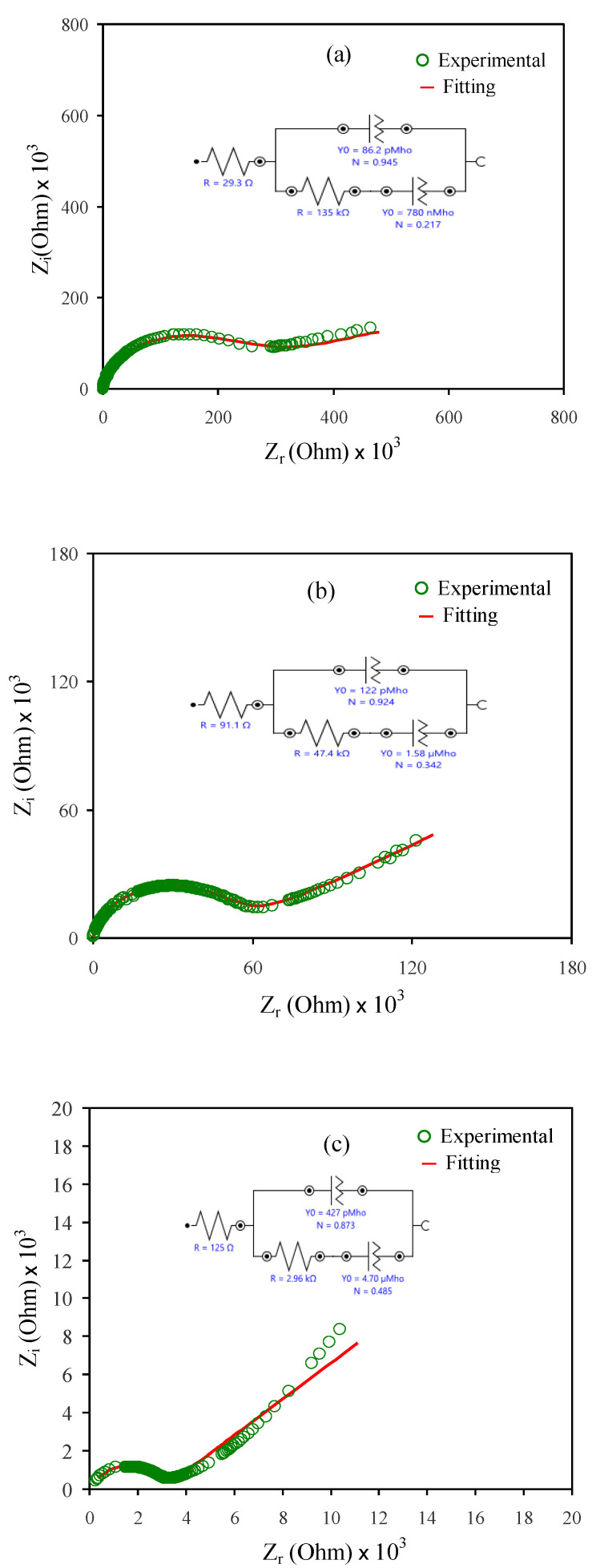

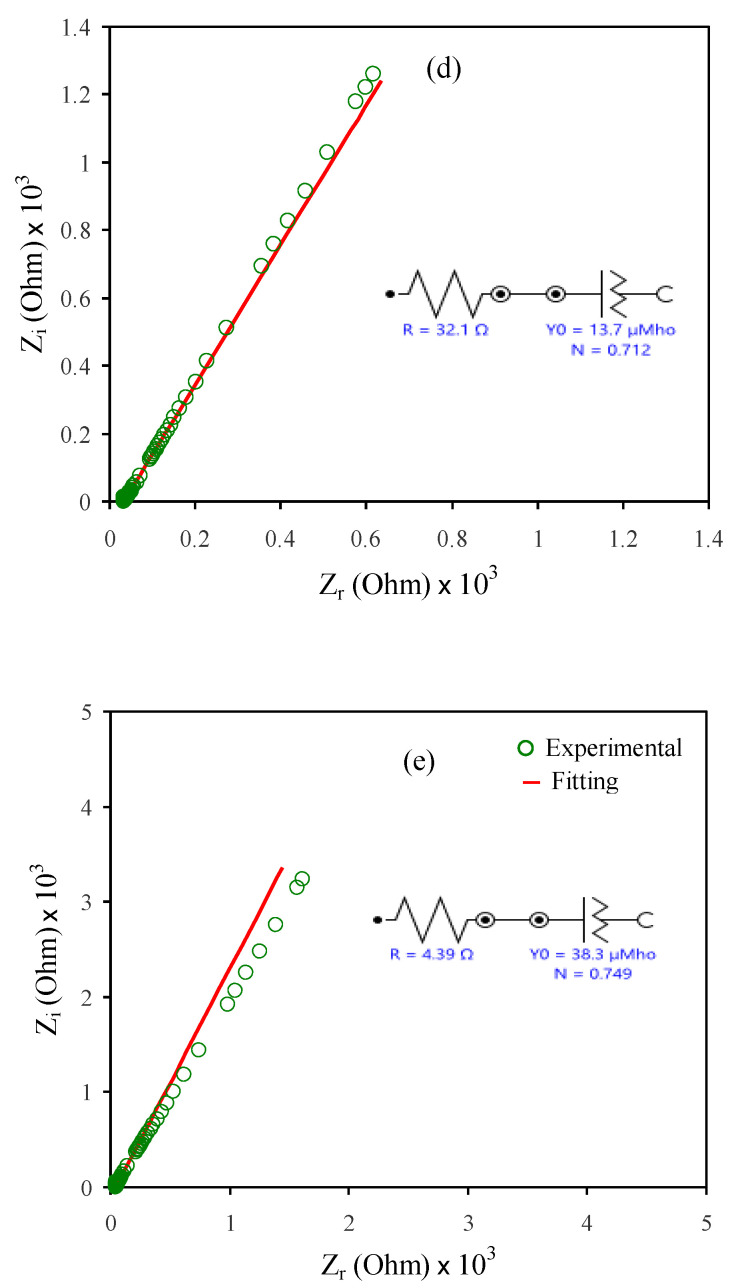
EIS plots for (**a**) MCSPE1, (**b**) MCSPE2, (**c**) MCSPE3, (**d**) MCSPE4, and (**e**) MCSPE5 electrolyte films.

Complex impedance spectroscopy (CIS) is one of most suitable techniques for studying the dynamics of ions within the PE matrices. The measurements of impedance clarify the conductivity behavior of the SPEs in the range of frequency [35]. The Cole–Cole profile s(Nyquist plots) of MC:NaI-based PEs at room temperature are exhibited in Figure 4a–e. It can be obviously seen that there was a semicircle and a spike in the high and low frequency ranges, respectively, at a low salt concentration. The two responses in the high and low frequency ranges can be explained on the basis of the phenomena that occur during electrochemical impedance perturbation. The semicircle at the high frequency region was caused by a parallel combination of bulk resistance (*R_b_*) and bulk capacitance, resulting from ion migration and immobile polymer chains, respectively. On the other hand, the spike at the low frequency region resulted from polarization, which occurs at the electrode/electrolyte interfacial region [18].

In Figure 4a-e, N is the deviation of the semicircle radius from the imaginary axis and the deviation of the tail/spike from the real axis. R and Y_0_ are the resistance and constant phase element capacitance, respectively. The centers of the semicircles lie below the *Z_r_*-axis, indicating the non-Debye behavior of ion relaxation. It was noted that the diameter of the semicircle at the high frequency region decreased as the concentration of NaI increased. It was also noticed that at 40 and 50 wt.% of NaI, the semicircle disappeared. This is due to relatively high resistance within the polymer matrix where mobile ion is responsible for conduction [33,36]. The *R_b_* of the electrolyte samples decreased with increasing NaI concentration.

Electric equivalent circuit (EEC) modeling is required for the fitting process, data analysis, and to show a comprehensive electrochemical image of the system [37]. The impedance spectra can be inferred in terms of equivalent circuit, for example, there is *R_b_* response as a result of the dynamics of the charge carrier and two constant phase elements (CPEs), as shown in Figure 4a–c. In the high frequency region, *R_b_* and CPE are responses of the system to the electrochemical perturbation. In the low frequency region, there was only a CPE as a result of the development of double-layer capacitance between the electrode and SPE. The term constant phase element (CPE) is used in the equivalent circuit in the place of an ideal capacitor in real systems (real system consists of two conductive electrodes separated by an insulator). In other words, CPE is used for a pseudo-capacitor where a conductor electrode is in contact with an electrolyte containing ions [38].

The impedance *Z_CPE_* can be mathematically expressed as follows [39,40]:

(4) $$\begin{array}{l} Z_{CPE}=\frac{1}{Q_{}\omega^{n_{}}}e^{-j\frac{\pi }{2}n}=\frac{1}{Q_{}\omega^{n_{}}}[\cos(\frac{n\pi }{2})-j\sin(\frac{n\pi }{2})],\\ 0\leq n\leq 1 \end{array}$$

where the CPE capacitor is denoted by *Q*; *ω* is the angular frequency; and *n* is the factor in relation to the nonconformity of the vertical axis of the complex impedance spectra.

Finally, the total impedance (*Z_total_*) related with the equivalent circuit (insets of Figure 4a–c) can be expressed as:

(5) $$Z_{total}=(R_{1}+\frac{A^{\prime }}{A^{\prime 2}+A^{\prime \prime 2}})-j(\frac{A^{\prime \prime }}{A^{\prime 2}+A^{\prime \prime 2}})$$

In Equation (5),

(6) $$A^{\prime }=Q_{1}\omega^{n_{1}}\cos(\frac{n_{1}\pi }{2})+\frac{B^{\prime }}{{B^{\prime }}^{2}+{B\prime \prime }^{2}}$$

(7) $$A^{\prime \prime }=Q_{1}\omega^{n_{1}}\sin(\frac{n_{1}\pi }{2})+\frac{B^{\prime \prime }}{B^{\prime 2}+{B\prime \prime }^{2}}$$

In Equations (6) and (7),

(8) $$B^{\prime }=R_{2}+\frac{1}{Q_{2}\omega^{n_{2}}}\cos(\frac{n_{2}\pi }{2})$$

(9) $$B^{\prime \prime }=\frac{1}{Q_{2}\omega^{n_{2}}}\sin(\frac{n_{2}\pi }{2})$$

All circuit elements used for the fitting process of the experimental impedance spectra for all the samples are presented in Table 3. It is well-defined that in the Cole–Cole plot, at a certain high concentration of the salt (Figure 4d,e), the semicircle disappears, which indicates the predominance of the resistive component of the polymer systems [41]. The mathematical expression of *Z_total_* is shown below:

(10) $$Z_{total}=[R_{1}+\frac{1}{Q_{}\omega^{n_{}}}\cos(\frac{n\pi }{2})]-j[\frac{1}{Q_{}\omega^{n_{}}}\sin(\frac{n\pi }{2})]$$

To calculate the ionic conductivity (σ) of SPEs, Equation (1) can be applied. It can be noted that there is a decrease in *R_b_* when the concentration of NaI is increased as a consequence of increasing the number of charge carriers [42]. For example, Table 4 shows the effect of NaI concentration on the DC conductivity of SPE. It is intuitive that both the number of charge carriers and charge carrier mobility contribute to the whole conductivity at ambient temperature via the equation below [43,44,45]:

*σ* = Σ *n_i_q_i_µ_i_* (11)

where *n_i_* is the charge carrier density; *q_i_* is equal to 1.6 × 10^−19^ C; and *µ_i_* is the ion mobility. In general, based on Equation (11), the number of charge carriers and mobility influence the conductivity [20]. Based on the facts previously stated, such sharp DC conductivity increasing at 50 wt.% can be ascribed to the increasing number of charge carriers [33,43]. Meanwhile, the XRD results confirmed the increase in the amorphous domain, resulting in increased ion mobility.

### 4.3. Dielectric Properties and Electric Modulus Study

#### 4.3.1. Dielectric Study

**Figure 5:**
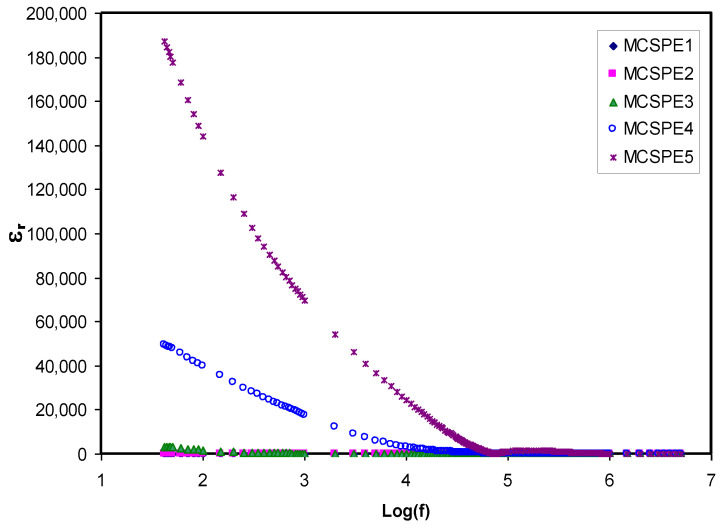
Dielectric constant versus log (f) for all electrolyte samples.

**Figure 6:**
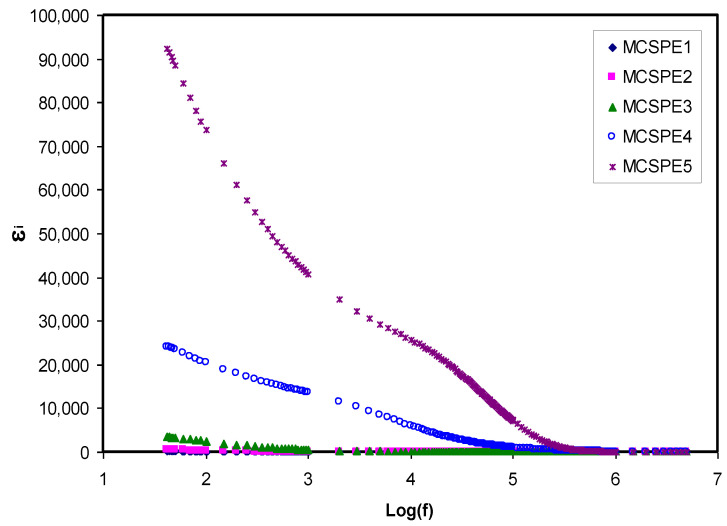
Dielectric loss versus log (f) for all electrolyte samples.

Dielectric spectroscopy is used in measuring the dielectric properties of the SPEs as a function of frequency. The principle of this technique is based on the interaction between an electric dipole body and external field. This interaction results in a response over a wide range of frequencies that appears in an impedance spectrum. Figure 5 and Figure 6 reveal the extent of the dependency of the dielectric constant and dielectric loss on frequency for various NaI salt concentrations at room temperature. The dielectric constant and dielectric loss can be obtained from the real (*Z_r_*) and imaginary (*Z_i_*) part of the complex impedance (Z^*^) using the relationship shown below [46,47]:

(12) $$\varepsilon_{r}=\frac{Z_{i}}{\omega \;C_{{}^{\circ}}(Z_{r}{}^{2}+Z_{i}{}^{2})}$$

(13) $$\varepsilon_{i}=\frac{Z_{r}}{\omega C_{{}^{\circ}}(Z_{r}{}^{2}+Z_{i}{}^{2})}$$

where *C_o_* has the usual meaning (vacuum capacitance) and is equal to *ε_o_A/t*; *ε_o_* is the permittivity of free space (8.85 × 10^−12^ F/m); *ω* is the angular frequency (*ω* = *2πf*); and *f* is the applied field frequency. From Figure 5 and Figure 6, it can be seen that in the low frequency region, there was dispersion of both the dielectric constant and dielectric loss spectra that also accounted for the large dielectric constant and dielectric loss obtained. This was due to the occurrence of space charge polarization at the electrode/electrolyte interfacial region [48]. Furthermore, the low frequency provides sufficient time for dipoles and charge carriers to orient their directions under an applied electric field. Moreover, electrode polarization results in charge carrier growth at the electrode/electrolyte interface. This phenomenon suppresses dielectric properties (bulk property) at a high frequency [47,49,50,51]. Aziz [47] also noted these variations where there was high dielectric constant in the region of low frequency as a result of the charge carrier accumulation or polarization effect near the electrodes. However, the *ɛ_r_* and *ɛ_i_* values (see Figure 5 and Figure 6) decreased with increasing frequency until it reached constant values at high frequency because the periodic reversal of the applied electric field occurs very quickly. In respect of energy storage, charge buildup at the electrolyte and electrode interface results in the electric dispersion in the intermediate frequency region [44,45].

#### 4.3.2. Electric Modulus Studies

The real (*M_r_*) and imaginary (*M_i_*) parts of the complex electric modulus (M^*^) can be computed using Equations (14) and (15):

(14) $$M_{r}=[\frac{\varepsilon_{r}}{\left(\varepsilon_{r}{}^{2}+\varepsilon_{i}{}^{2}\right)}]=\omega C_{o}Z_{i}$$

(15) $$M_{i}=[\frac{\varepsilon_{i}}{\left(\varepsilon_{r}{}^{2}+\varepsilon_{i}{}^{2}\right)}]=\omega C_{o}Z_{r}$$

**Figure 7:**
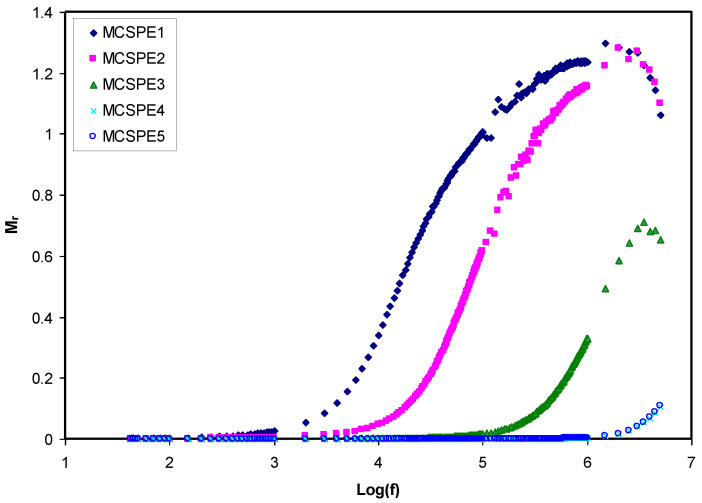
Real part of the electric modulus versus log (f) for all electrolyte samples.

**Figure 8:**
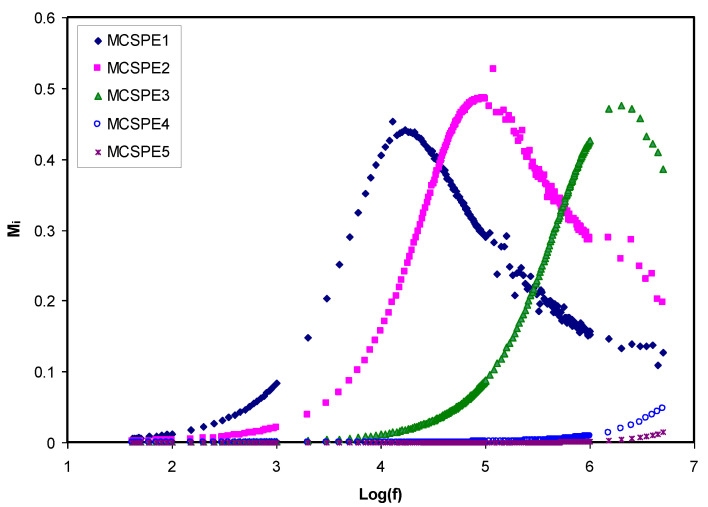
Imaginary part of the electric modulus versus log (f) for all electrolyte samples.

Figure 7 and Figure 8 show *M*_r_ and *M_i_* versus frequency for the electrolyte films at room temperature. *M*_r_ and *M_i_* decrease with extended tails in the low frequency region, meaning that the electrode polarization will create a negligible participation. The electrode polarization, which causes the buildup of charges at the stainless steel electrodes, is suppressed [52]. The *M*_r_ and *M_i_* spectra show different behavior in comparison with the pattern of *ε_r_* and *ε_i_*. The large *ε_r_* and *ε_i_* values (see Figure 5 and Figure 6) were seen at low frequency. Normally, the *M*_r_ and *M_i_* in M^*^ are created as a result of the inverse of *ε_r_* and *ε_i_* in ε^*^, respectively. The *M*_r_ and *M_i_* indicate the lowest values in the low frequency region, which indicate the material capacitive behavior.

In the low frequency region, extended tails were seen for the electrolyte films in Figure 7 and Figure 8. This could be ascribed to the suppression of the electrochemical double-layer at the electrode/electrolyte films as the result of the large value of capacitance in the area of low frequencies. It is vital to observe that *M*_r_ obtained the highest level of saturation in the high frequency region. This is because *ε_r_* at high frequencies decreased to the lowest value and as a result, *M*_r_ increased to the maximum [47]. In comparison to the spectra of *ε_i_* (Figure 6), noticeable peaks as the result of the conductivity relaxation were seen in the *M_i_* spectra of the MCSPE1, MCSPE2, and MCSPE3 electrolyte films (see Figure 8). In previous work, it was shown that the *ε_i_* is affected by means of an ohmic conduction (specifically DC conductivity), causing the absence of loss peaks in the spectra of *ε_i_* (see Figure 6) [53].

### 4.4. Electrochemical Studies

#### 4.4.1. Determination of Ionic Transference Number

**Figure 9:**
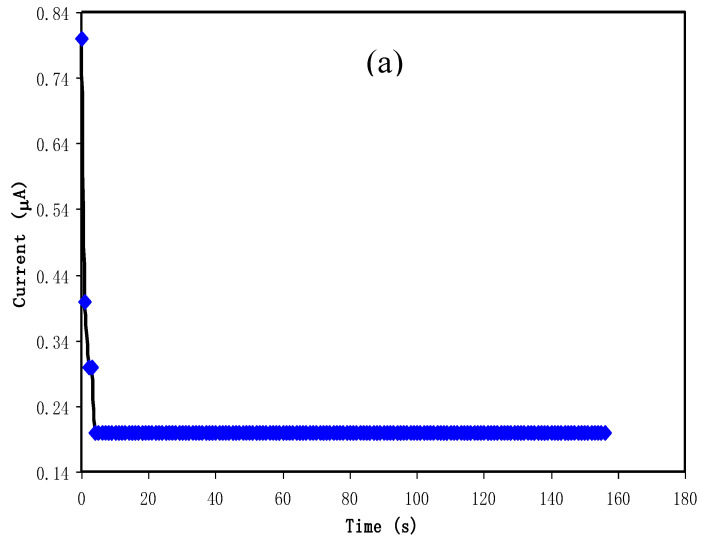

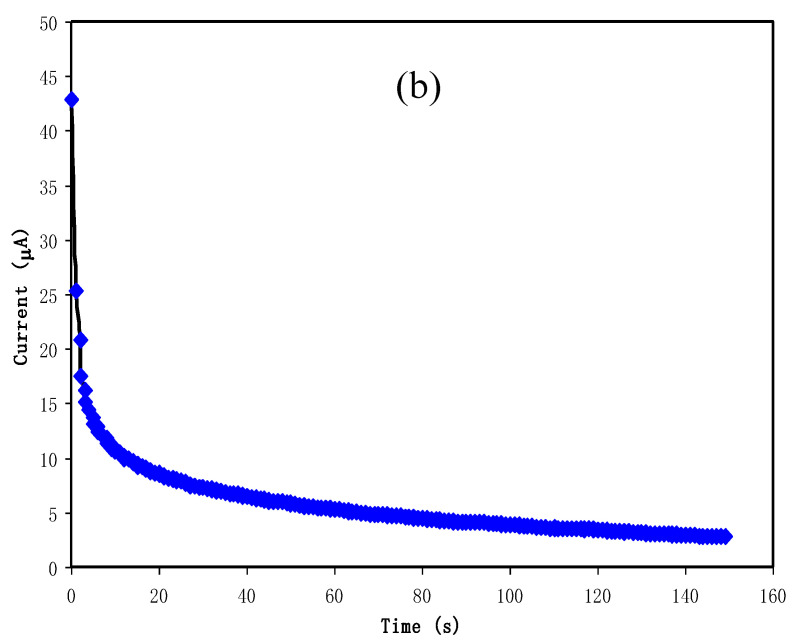
Current versus time for the (**a**) MCSPE4 and (**b**) MCSPE5 electrolyte systems.

The polarization plot of electrolyte with (a) MCSPE4 and (b) MCSPE5 are shown in Figure 9a,b. Commonly in a PE, the total conductivity is through both the contribution of electron and ions. For the EDLC fabrication purpose, *T_ion_* must be bigger than *T_elec_*, thus TNM analysis was conducted at 0.2 V. The polarization of SS|MCSPE4|SS and SS|MCSPE5|SS are shown in Figure 9a,b, respectively. The starting current (*I_s_*) of MCSPE4 was 0.80 μA and the balanced current (*I_b_*) was 0.20 μA. These values were lower compared to when 50 wt.% NaI was added. As we obtained the value of *I_s_* and *I_b_*, *T_ion_* can be determined using the following equation:

(16) $$T_{ion}=\frac{I_{s}-I_{b}}{I_{s}}$$

where the summation of *T_ion_* with *T_elec_* is equal to 1. It was discovered that the inclusion of 40 wt.% NaI provided 0.75 while for 50 wt.%, NaI was 0.93. As additional salt is introduced, more solvated ions are produced. Thus, more ions can be conducted toward SS electrodes and form a double-layer. Hence, it resulted in a high ionic transference number. It is also noticeable in Figure 9a,b that the current was high at the beginning, which is at this point that both ions and electrons are conducted. As time goes by, ions are blocked at the surface of the electrodes and at this point, the cell is polarized whereas the current flows are due to electrons.

#### 4.4.2. Determination of Electrolyte Potential Stability

**Figure 10:**
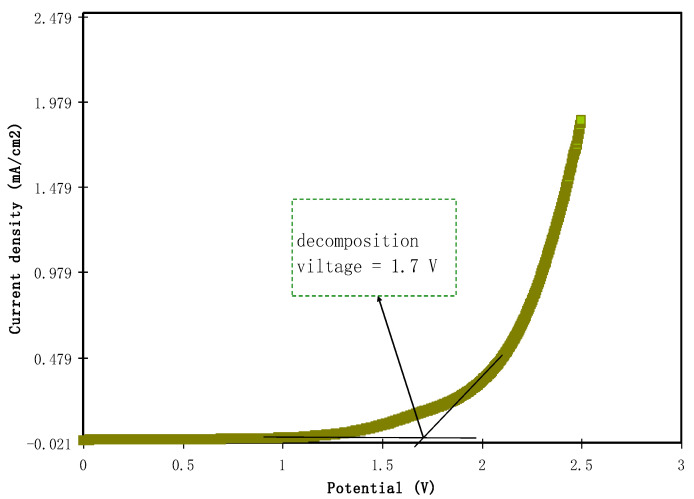
LSV plot of the highest conducting electrolyte.

In energy storage technologies, the potential constancy of the electrolyte is a crucial determination. The maximum potential limit of the electrolyte or the potential where the electrolyte starts to degrade can be obtained using LSV analysis. Figure 10 illustrates the LSV for the SS|highest conducting electrolyte (MCSPE5)|SS at 10 mV/s. The current was observed to be constant at the potential <1.50 V. As the potential elevates more than 1.50 V, current underway grows sharply. To obtain the value of the decompose voltage, a straight line was drawn with respect to the constant current and sharp current increase. The point of intersection was the decompose voltage, which is in this case was 1.70 V. This value is good enough for the application of proton-based energy devices; typically an electrolyte should have at least a decomposition voltage of 1 V.

#### 4.4.3. CV Studies of Fabricated EDLC

**Figure 11:**
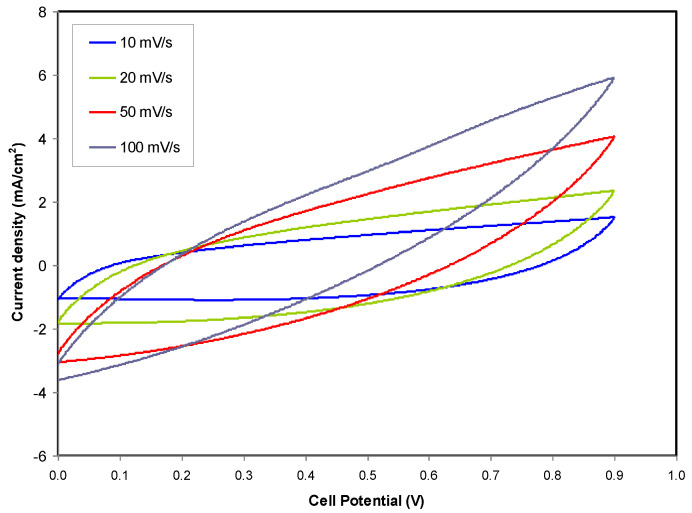
CV plots of the fabricated EDLC at different scan rates.

The fabricated EDLC was charged–discharged using CV analysis and the CV plot can be seen in Figure 11. CV analysis is well known as a tool to confirm the capacitive behavior of an EDLC. It is obvious that for all scan rates, the CV plots have no redox peaks. This means that the mechanism of energy storage in this EDLC is through the adsorption/desorption of ions at the surface of AC electrodes [54]. The shape of the CV plot at 100 mV/s is leaf-like shape, while the shape changes to a more rectangular shape as the scan rate decreases to 10 mV/s. Fattah et al. [54] stated that the rectangular CV plot possessed excellent electrochemical stability. Current is independent of potential for rectangular CV, which means that more polarization or charge double-layers occur in the EDLC. The rectangular CV also showed a close mirror image symmetry of the current replies to the zero line, which settles the electric double-layer capacitive behavior. Table 5 shows the parameters extracted from the CV equation. The value of specific capacitance (*C*) for 100 mV/s was 15.8 F/g. The value of *C* seemed to increase as the scan rate was reduced to 10 mV/s. Constant ionic migration toward the AC electrodes is possible at a low scan rate. This condition provides much more polarization, thus providing a higher *C* value. As the scan rate increases, ions move in a very fast motion, which leads to improper charge double-layer development [55]. The decrement in *C* with an increase in the scan rate is interconnected with the existence of internal resistance. The time scale of the current to grasp a horizontal constant value on setback of the potential scan is amplified at a high scan rate. The longer postponement at the switching potential encourages the slow restructuring of the electrical double-layer due to the high resistance of ionic mobility in the micro-pores of activated carbon in EDLC [56,57]. Table 6 shows various AC-based EDLC with different electrolyte materials.

## 5. Conclusions

Summarily and descriptively, green SPE of methyl cellulose (MC) incorporated with various concentrations of NaI were prepared using the solution cast method. The structural studies confirmed the disruption of the crystalline phase with various degrees and the formation of an amorphous phase by salt addition of different concentrations. The conversion of the crystalline phase to the amorphous phase also caused a lowered bulk resistance to a large extent, which was emphasized from the impedance spectra (*Z_i_* vs. *Z_r_*). The highest achievable DC conductivity was 3.01 × 10^−3^ S/cm at 50 wt.% of NaI at room temperature. From thee dielectric analysis, all prepared samples were non-Debye behavior, as evidenced from the asymmetric broad peaks in the *M_i_* spectra. In the MC–NaI system, the primary charge carrier was ions. Significant enhancement of DC ionic conductivity was obtained when the highest concentration of NaI changed the ion transference number by 14%. The mechanism of charge storing in the EDLC assembly is the capacitive process (non-Faradaic process). The specific capacitance of the EDLC design is influenced by the scan rate, where it is higher at a low scan rate.

## Figures and Tables

**Table 1:** Composition of MC:NaI-based solid polymer electrolytes.

Designation	MC (g)	NaI Wt.%	NaI (g)
MCSPE1	1	10	0.111
MCSPE2	1	20	0.250
MCSPE3	1	30	0.428
MCSPE4	1	40	0.666
MCSPE5	1	50	1

**Table 2:** The *Xc* using deconvoluted XRD examination.

Electrolyte	Degree of Crystallinity (%)
MCSPE1	22.38
MCSPE2	18.18
MCSPE3	17.43
MCSPE4	14.08
MCSPE5	10.66

**Table 3:** The EEC fitting parameters for MC:NaI systems at room temperature.

Fitting Circuit	R(Q(RQ))	R(Q(RQ))	R(Q(RQ))	RQ	RQ
Sample	MCSPE1	MCSPE2	MCSPE3	MCSPE4	MCSPE5
*R_b_* (Ω)	134,659.276	47,476.077	3084.850	32.124	4.385
*R_s_* (Ω)	29.276	91.077	125.250	32.124	4.385
*Q*_1_ (F)	8.62 × 10^−11^	1.22 × 10^−10^	4.27 × 10^−10^	1.37 × 10^−5^	3.8 × 10^−5^
N_1_	0.945	0.924	0.873	0.712	0.749
*R*_2_ (Ω)	134,630.0	47,385.0	2959.6	-	-
*Q*_2_(F)	7.80 × 10^−7^	1.58 × 10^−6^	4.70 × 10^−6^	-	-
N_2_	0.217	0.342	0.485	-	-
X_2_ (Ω)	0.472	0.230	0.503	10.732	66.927

**Table 4:** DC conductivity for MC:NaI systems at room temperature.

Designation	Conductivity (S cm^−1^)
MCSPE1	9.95 × 10^−8^
MCSPE2	2.69 × 10^−7^
MCSPE3	4.33 × 10^−6^
MCSPE4	4.12 × 10^−4^
MCSPE5	3.01 × 10^−3^

**Table 5:** Parameters extracted from the CV equation.

Scan Rate (mV/s)	Capacitance (F/g)
100	15.8
50	32.4
20	65.5
10	94.7

**Table 6:** AC-based EDLC fabricated and reported using different electrolyte materials.

System	*C* From CV (F/g)	Reference
PVA–NaTf PVA–NaTf–BmImBr	~10.0 (at 10 mV/s) ~12.0 (at 10 mV/s)	[58]
PVA–LiClO_4_	10.9 (at 10 mV/s)	[59]
HEC–MgTf_2_–EMIMTf	25.0 (5 mV/s)	[60]
PMMA–Mg(CF_3_SO_3_)_2_)	27.0 (5 mV/s)	[61]
PAA–LiTFSI–TiO_2_	28.5 (10 mV/s)	[62]
Corn starch–LiOAc	33 (0.5 mV/s)	[63]
CS:MC:NH_4_F	58.3 (10 mV/s)	[64]
PEO:NH_4_SCN: CeO_2_	86.9 (20 mV/s)	[65]
PVA–KOH	99 (10 mV/s)	[66]
PVA:NH_4_SCN:Ce(III)–complex:gly	108.37 (10 mV/s)	[25]
MC–NaI	94.7 (at 10 mV/s)	This work
